# Identification and Ablation of an Incidental Concealed Accessory Pathway During Atrial Fibrillation Ablation

**DOI:** 10.19102/icrm.2023.14113

**Published:** 2023-11-15

**Authors:** Dhruv Rajpurohit

**Affiliations:** 1 Ascension Providence Hospital, Southfield, MI, USA

**Keywords:** Ablation, accessory pathway, atrial fibrillation, mapping

## Abstract

A 69-year-old man with persistent atrial fibrillation (AF) experiencing recurrences of suspected paroxysmal atrial tachycardia was referred for repeat ablation. He had previously undergone pulmonary vein isolation in 2016. He was suspected to be experiencing a tachycardia involving the mitral isthmus or left-sided veins initially; however, electroanatomic mapping did not reveal a circuit involving these structures. Instead, a focal arrhythmia with the earliest signal on the anterolateral mitral valve annulus was noted. Catheter manipulation in this region consistently terminated the tachycardia.

## Case presentation

**Figure 1:**
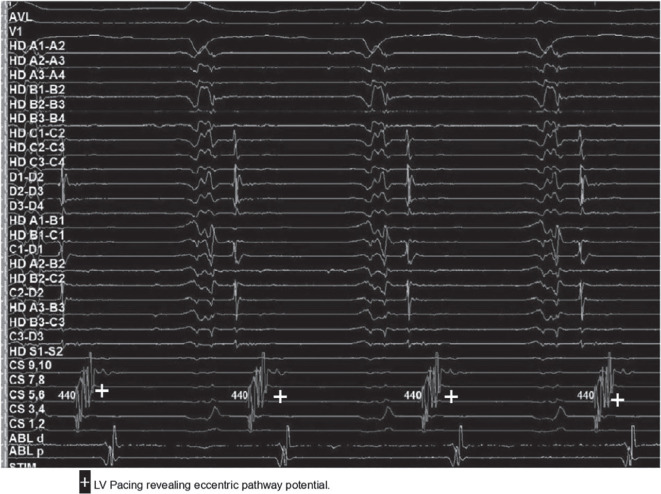
Electrograms revealing eccentric tachycardia.

**Figure 2:**
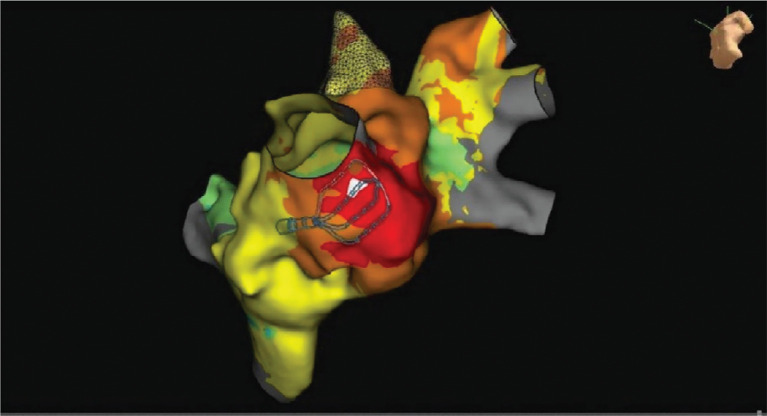
Electroanatomic map created with HD Grid catheter. The earliest left atrial signal, recorded during left ventricular pacing, is indicated by the white color.

**Figure 3:**
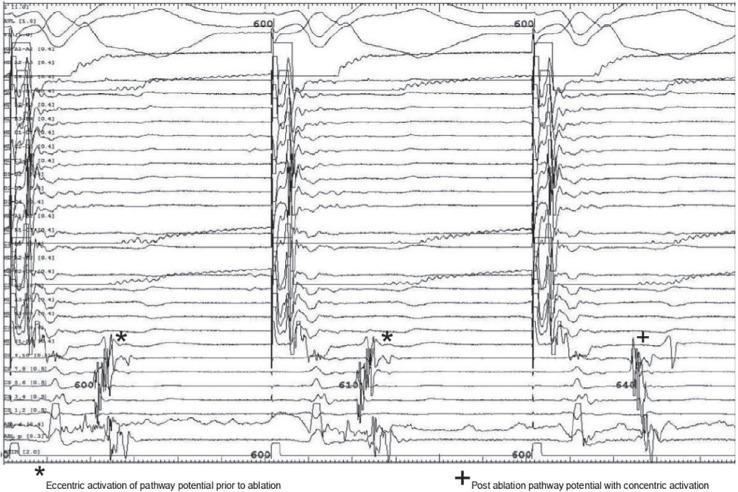
Electrogram revealing elimination of pathway potential on ablation catheter resulting in change from eccentric to concentric activation in the coronary sinus with left ventricular pacing.

A 69-year-old man with persistent atrial fibrillation (AF) status post-ablation with pulmonary vein isolation in 2016 with recurrences of suspected paroxysmal atrial tachycardia was referred for repeat ablation. Electroanatomic mapping (EAM) in the left atrium spontaneously induced supraventricular tachycardia (SVT). The right pulmonary veins were ostially isolated and the left pulmonary veins had recovered at the anterior carina. The Advisor™ HD Grid Mapping Catheter, Sensor Enabled™ (Abbott, Chicago, IL, USA) and EnSite Precision™ (Abbott) three-dimensional mapping system were used to map this 1:1 eccentric atrioventricular tachycardia with a cycle length of 430 ms **(Figure 1)**. A tachycardia involving the mitral isthmus or left-sided veins was suspected initially; however, EAM did not reveal a circuit involving these structures. Instead, a focal arrhythmia with the earliest signal on the anterolateral mitral valve annulus was noted **(Figure 2)**. Catheter manipulation in this region consistently terminated the tachycardia. The Advisor™ HD Grid was then placed in the left ventricle just distal to this site, and although left ventricular (LV) pacing resulted in termination, eccentric activation was confirmed. Mapping with the ablation catheter during LV pacing revealed a pathway potential, which was eliminated instantly with ablation, restoring concentric activation **(Figure 3)**.

## Discussion

AF is noted to occur in up to one-third of patients with accessory pathways (APs). There is a 15 times higher incidence of AF occurring in patients with manifest pathways compared to those with concealed accessory pathways (CLAPs), as the overall prevalence of AF in CLAP patients is thought to be <1%.^1^ The mechanisms and pathogenesis of AF in patients with APs are poorly understood, complex, and often controversial. Even though therapeutic ablation of APs is associated with AF reduction, older age, male sex, and the presence of APs are independent risk factors for the presence and progression of clinical AF.^2^ Numerous additional risk factors like hypertension, diastolic heart failure, older age, complexity of coronary artery disease, structural changes in the atrium and ventricle, and the presence of renal disease all contribute to the development of AF in patients with CLAPs.^3^ Given the presence of these risk factors in our patient, it is likely that APs are only one of the many substrates for the development of AF, which was unlikely related to his AP.^3^ Additionally, previous studies have documented an AF recurrence rate of 10%–25% following AP ablation, and close monitoring of these patients is necessary for uncovering future recurrences.^1^ This case serves as a reminder to consider the differential of SVT in cases of redo AF ablations and revealed the likely therapeutic advantage of LV pacing near the pathway site to delineate a clear pathway potential.^4,5^
